# Caregivers' perceptions, knowledge, and perceived barriers to pediatric procedural pain management: a cross-sectional study

**DOI:** 10.1097/PR9.0000000000001460

**Published:** 2026-07-31

**Authors:** Meredith Otley, Christine T. Chambers, Jennifer A. Parker, Isabel Jordan, Kirstin Weerdenburg, Justine Dol

**Affiliations:** a IWK Health, Halifax, Canada; b Undergraduate Medical Education, Faculty of Medicine, Dalhousie University, Halifax, Canada; c Department of Psychology and Neuroscience, Dalhousie University, Halifax, Canada; d Department of Pediatrics, Dalhousie University, Halifax, Canada; e Emergency Medicine, IWK Health, Halifax, Canada; f Department of Emergency Medicine, Dalhousie University, Halifax, Canada

**Keywords:** Procedural pain, Acute pain, Caregivers, Pediatrics, Pain management

## Abstract

**Introduction:**

Pain from medical procedures can have negative effects on children and adolescents (eg, healthcare avoidance), and is often not adequately prevented and treated. Best practices emphasize the important role of caregivers in procedural pain management.

**Objectives:**

To investigate caregivers' perceptions, knowledge, and behaviours related to their children's procedural pain management and to describe the barriers perceived by caregivers encountered during the management of their child's procedural pain.

**Methods:**

This was a cross-sectional online survey of caregivers with children aged 0 to 17 years. Statistical analysis of closed-ended responses and inductive content analysis of open-ended responses were completed.

**Results:**

Among 104 caregivers (56.7% female, average child age = 8.9 years), 93% were present during their child's recent painful procedure, 62% reported participating in pain management, and 66% perceived their child's recent procedural pain was well-managed. The most used pain management strategies reported were psychological (eg, 59% used deep breathing). Caregivers reported seeking information about pain management from electronic sources (37%) and/or healthcare providers (35%), and 70% reported being satisfied with the information available to them. Caregivers reported their own lack of knowledge, their child's emotions, communication breakdowns, and healthcare professional attitudes as barriers to helping with pain management.

**Conclusions:**

Caregivers are often present during their children's painful procedures, but many reported not being involved in pain management. Despite significant progress in procedural pain management, there remain knowledge gaps among caregivers and barriers to their active engagement. Addressing these may help to improve pediatric procedural pain management implementation.

## Abstract 2

Cross-sectional online survey of caregivers highlights self-reported knowledge gaps in best practices and perceived barriers to their active involvement in their child's procedural pain management.

## 1. Introduction

Procedural pain is defined as pain experienced by children as part of medical interventions, including needle injections (eg, vaccination, steroid, or insulin injections), blood draws, sutures, or urethral catheterizations.^38^ Children undergo painful medical procedures throughout their lifetime as part of regular and emergent medical care, chronic medical conditions, and disease management. Infants and children receive more than 20 vaccinations at their doctor's office and school as part of the recommended Canadian immunization schedule.^29^ Hospitalized children also undergo a high frequency of painful procedures, with a study finding that 78.2% of hospitalized infants and children had undergone at least one painful procedure in a 24-hour period.^4,32^ Procedural pain is also among the most common sources of acute pain in pediatric emergency departments.^27^

Despite procedural pain being common for children, not all children receive appropriate pain management. Access to pain management is a human right as affirmed by the International Association for the Study of Pain in the Declaration of Montreal.^18^ As was suggested by the Lancet commission,^9^ initiatives are currently working to move pediatric pain research into practice, such as the Pediatric Pain Management Health Standard codeveloped by Solutions for Kids In Pain and the Health Standards Organization.^28^ Even with available best practice statements for procedural pain management in children, including those from the Canadian Paediatric Society (CPS)^38^ and guidelines for specific types of procedural pain, such as vaccination-related pain,^33^ procedural pain remains undertreated.^4,31,35^ For example, despite guidelines advocating for topical lidocaine use for procedural pain management, only 29% of pediatric patients undergoing needle procedures at a Canadian hospital received it over a 24-hour period.^31^ Time and staffing constraints, staff culture (eg, lack of prioritization of pain management), and staff knowledge gaps have previously been identified by healthcare providers as barriers to implementation of adequate procedural pain management.^1,17^ Poorly managed procedural pain is associated with negative immediate effects (eg, distress, physiological consequences) and long-term effects (eg, pain sensitization, fear of needles, health care avoidance).^3,20,33–35,37^ A meta-analysis from 2022 found that the prevalence of injection pain and needle fear as a barrier to vaccination was 5% to 13%.^36^ Thus, there is a need to improve pediatric pain management implementation.

To improve the implementation of best practices in pediatric pain management, consideration of the caregivers' role is essential. Caregivers are often present when their children experience procedural pain, providing a unique opportunity for them to serve as coaches and advocates. In a systematic review of parent experiences witnessing their children's procedural pain, qualitative data showed that they valued taking an active role in pain management.^14^ There is evidence that supporting parents to take this active role in their child's care is beneficial^39^ and CPS position statement affirm that doing so is essential for effective procedural pain management.^38^ The Canadian Pediatric Pain Management Standard states that lack of accessible resources and training for caregivers is a barrier to implementation of evidence-based strategies.^28^ There are existing initiatives that target parents to improve the implementation of procedural pain management best practices including the “Be Sweet to Babies” campaign for newborn screening, vaccinations, and other painful procedures,^2^ the “It Doesn't Have To Hurt” video for needle pain and distress in children and #ItDoesntHaveToHurt social media initiative on children's pain management,^6,7^ and the CARD (Comfort-Ask-Relax-Distract) system for childhood vaccinations.^33^ It is important to learn about caregivers' current behaviours related to procedural pain management, their perceptions of their role in it, and the extent to which they are interacting with existing pediatric procedural pain management resources. It is also important to understand the challenges caregivers encounter when attempting to help with their children's pain management, to be able to support them in improving implementation of best practices.

Therefore, the objectives of this study were to:

(1) Investigate caregivers' perceptions, knowledge, and behaviour related to their child's procedural pain management. It was hypothesized that some caregivers would not be satisfied with, fully informed about, or actively involved with their child's procedural pain management.
(2) Describe the perceived barriers caregivers experience related to helping manage their child's procedural pain. As this objective used open-ended questions that were analyzed qualitatively, no hypothesis was made.

## 2. Methods

### 2.1. Study design

A large cross-sectional online survey was conducted that focussed on pediatric procedural pain management but contained 2 studies each with distinct aims. The data assessed in this study is focussed on caregivers' perceptions, knowledge, behaviours, and perceived barriers to their children's procedural pain management, variables that speak to current practice and modifiable determinants of care. The data from the complementary aim will be presented in a separate article focussed on the need for and potential features of a procedural pain management knowledge mobilization intervention for caregivers, data that speak to intervention design requirements, not current behaviour.^24,25^ The present and concurrent articles share the same sampling frame, recruitment procedures, and analytic approach, but analyze nonoverlapping variables and item sets (ie, no overlapping analysis of response items) and address different a priori aims; no results are duplicated.

### 2.2. Setting and sample

Caregivers who lived in Canada and had a child aged 0 to 17 years were eligible to participate. Caregivers were defined as those who held direct childcare responsibility for a child in this age range regardless of the biological relationship (eg, parents, grandparents, and guardians). Caregivers also had to be able to read and write in English to take part in the English-language survey.

### 2.3. Data collection

Data collection was hosted on Qualtrics, a subscription-based online survey platform in September 2023. The survey took approximately 27 minutes to complete, as estimated by the Qualtrics survey software. Participants were recruited through engagement with Leger, a Canadian market research company, who sent out an email to a subset of their existing panel. Respondents first completed eligibility screening questions to ensure they met the inclusion criteria and then gave informed consent to participate in this study. To recruit a diverse sample of respondents, maximum quotas were set in Qualtrics to limit the number of respondents who met certain demographic characteristics (sex of their child, race of the child) corresponding to the population proportion from the Canadian 2021 government census.^15^ Thus, respondents with White children were limited to 73.6% of initial respondents and the respondents with male and female children were limited to 49.2% and 50.7%, respectively. Quotas also limited the number of respondents whose children were in 5 age groups (0–2, 3–6, 7–10, 11–14, and 15–17 years) to sample caregivers of children over the span of childhood development. Respondents who completed the online survey were compensated by Leger with a quantity of points that was in line with their policies for completing surveys of that length. Through Leger, points collected from this and other surveys could later be redeemed by respondents for rewards (eg, gift cards). This study was approved by the IWK Health Research Ethics Board (#1026213).

### 2.4. Measures

The survey had a mix of 55 closed- and 8 open-ended questions about the caregiver's demographic characteristics (eg, age, sex, gender, race, and household income), their child's demographic characteristics, and caregivers past experiences with their child's procedural pain. The questions about the respondent's demographic characteristics were formulated to align with current best practices on collecting race-based and gender identity data.^30^ Caregivers were first asked about their child's most recent procedure and then were asked about their child's experiences with procedural pain throughout their lifespan. Caregivers who had multiple children were asked to choose one to consider when responding to the survey questions. Closed-ended question responses included dichotomous “yes or no” questions, 5-point Likert-type scales (1 = *not at all* and 5 = *very much*) and checklist questions where respondents were able to select all responses that applied. Respondents were also given the opportunity to answer open-ended questions. Respondents were required to respond to every question by the survey software, but all questions included a “prefer not to answer” option. Respondents were informed that they were able to stop the survey at any time by exiting and any incomplete responses were excluded from analysis. The survey questions were researcher-generated and co-developed with a patient partner member of the research team (I.J.).

### 2.5. Data analysis

Because of the potential for invalid responses when collecting data through online surveys, the data were screened and then cleaned using a researcher-generated procedure before analysis in keeping with best practices outlined in the literature.^8,16,23^ As such, the data were reviewed by the first author (M.O.) in discussion with 2 coauthors (J.D., J.A.P.) and responses were excluded from the analysis when they met specific predetermined criteria. Surveys that included at least one answer that was determined to be a “red flag” resulted in the exclusion of that survey response data from analysis. These red flag criteria included: (1) not meeting eligibility criteria; (2) total survey completion time of less than one third of the average completion time (<6.3 minutes); (3) multiple responses from identical IP addresses; (4) incomplete surveys; (5) answering honeypot questions, which are questions hidden to human respondents but visible to bots; and (6) nonsensical answers to open-ended questions (eg, “*I'm going to go ahead with the rest today I just wanted to make a quick reminder*”). Several additional criteria were designated as “yellow flags” meaning that if 2 or more answers on the survey met any of these criteria their responses would be excluded. These yellow flag criteria included: (1) total response time of less than one third of the estimated completion time from Qualtrics and pilot surveys (<9 minutes), (2) an IP address that was different from their indicated province of residence, and (3) incorrect answers to attention check questions (eg, “*The question you are about to answer is straight forward, when asked your favourite colour you must select* “orange.” *This is an attention check. Based on the text you read above, what is your favourite colour?*”)

Quantitative data were analyzed using the IBM Statistical Package for Social Sciences (SPSS 28). Descriptive statistics were used to characterize demographic data. Frequencies, means, and standard deviations were computed for all relevant outcomes. Mann–Whitney, Spearman Rho, and Kruskal–Wallis 1-way ANOVAs were used to explore differences between participants of diverse demographic groups or pain experiences and their perspectives on procedural pain management. Responses to open-ended questions were analyzed using the NVivo 14 program. Inductive content analysis was used, according to the process laid out by Elo and Kyngäs.^10^ The open-ended responses were reviewed and assigned unique codes which were then grouped into higher order categories. This analysis was led by the first author (M.O.) and verified by a second author (J.D.).

## 3. Results

### 3.1. Participants

A total of 140 respondents completed the survey, of which 36 respondents were excluded from the study, 30 for meeting red flag criteria and 6 for meeting 2 or more yellow flag criteria. Table 1 summarizes the demographic information for the participants (n = 104) that were included in the analysis and their children. Although quotas for the demographic variables were set in advance to guide data collection (as described in the methods), due to data cleaning, demographics differed from quotas as reported below. Caregivers were on average 39.6 years of age (SD = 7.0 years, range = 20–53 years) and their children were on average 8.9 years (SD = 5.1 years, range = 0–17 years).

**Table 1:** Demographic characteristics of caregivers and their children.

Demographic characteristics	Children (n = 104)	Caregivers (n = 104)
Child age		
Baby (0–12 mo)	5 (4.8%)	—
Toddler (1–2 y)	7 (6.7%)	—
Preschooler (3–4 y)	19 (18.3%)	—
School age (5–12 y)	38 (36.5%)	—
Adolescent (13–17 y)	35 (33.6%)	—
Sex at birth		
Female	54 (51.9%)	59 (56.7%)
Male	50 (48.1%)	45 (43.3%)
Gender		
Bigender	0 (0%)	1 (1.0%)
Boy/man*	47 (45.2%)	42 (40.4%)
Girl/woman†	51 (49.0%)	58 (55.8%)
Nonbinary	2 (1.9%)	0 (0%)
I don't identify with any option provided	2 (1.9%)	2 (1.9%)
Prefer not to answer	2 (1.9%)	1 (1.0%)
Province of residence		
Alberta	—	15 (14.4%)
British Columbia	—	18 (17.3%)
Manitoba	—	5 (4.8%)
New Brunswick	—	2 (1.9%)
Newfoundland and Labrador	—	2 (1.9%)
Nova Scotia	—	3 (2.9%)
Ontario	—	66 (63.5%)
Quebec	—	1 (1.0%)
Saskatchewan	—	2 (1.9%)
Race		
Single selection		
Black	5 (4.8%)	5 (4.8%)
East Asian	14 (13.5%)	13 (12.5%)
Latin American	2 (1.9%)	2 (1.9%)
Middle Eastern	2 (1.9%)	2 (1.9%)
South Asian	13 (12.5%)	15 (14.4%)
Southeast Asian	1 (1.0%)	1 (1.0%)
White	55 (52.9%)	60 (57.7%)
Not listed	1 (1.0%)	1 (1.0%)
Prefer not to answer	3 (2.9%)	1 (1.0%)
Multiple selections	8 (7.7%)	4 (3.9%)
History of hospitalization		
Yes	39 (37.5%)	—
No	65 (62.5%)	—
Fear of needles and/or needle phobia		
Yes	52 (50.0%)	22 (21.2%)
No	52 (50.0%)	82 (78.8%)
Frequency of medical procedures (eg, blood draws, injections, IV insertions)		
More than once per year	6 (5.8%)	—
Less than once per month but more than once per year	23 (22.1%)	—
Once per year	31 (29.8%)	—
Never/rarely	43 (41.3%)	—
Prefer not to answer	1 (1.0%)	—
Relationship to child		
Aunt or uncle	—	1 (1.0%)
Grandparent	—	1 (1.0%)
Guardian/caregiver‡	—	2 (1.9%)
Parent	—	100 (96.2%)
Education level		
Partial or completed high school	—	12 (11.5%)
Partial university (at least one year)	—	5 (4.8%)
Trade school/community college	—	21 (20.2%)
University graduate (4 y college)	—	48 (46.2%)
Graduate school/professional training	—	18 (17.3%)
Annual household income		
Less than $50,000	—	10 (9.7%)
$50,000–$100,000	—	36 (34.6%)
$100,000–$150,000	—	33 (31.7%)
$150,000–$200,000	—	10 (9.6%)
More than $200,000	—	10 (9.6%)
Prefer not to answer	—	5 (4.8%)
Current relationship status		
Divorced/separated	—	2 (1.9%)
Married/common-law	—	93 (89.4%)
Never married	—	9 (8.7%)

* Includes Boy-Cisgender (n = 37), Boy-prefer not to specify (n = 4), Boy-open text response (n = 6), Man-Cisgender (n = 34), Man-prefer not to specify (n = 6), Man-open text response (n = 2).

† Includes Girl-Cisgender (n = 44), Girl-prefer not to specify (n = 6), Girl-Transgender (n = 1), Woman-Cisgender (n = 52), Woman-prefer not to specify (n = 4), Woman-open text response (n = 2).

‡ Includes Guardian/Caregiver (n = 1), Foster Parent-open text response (n = 1).

### 3.2. Caregivers' perspectives, knowledge, and behaviours related to procedural pain

Caregivers were asked about their experience at their child's most recent painful procedure and their responses are summarized in Table 2. Most (91.9%) took place in a clinical setting (doctor's office, emergency room, inpatient setting) and 92.9% of caregivers were present with their child when the procedure took place. While 61.5% of caregivers indicated that they played a role in pain management, only 41.8% reported that healthcare providers provided pain management, and 6.6% reported no one managed their child's pain.

**Table 2:** Experiences of caregivers at their child's most recent painful medical procedure.

Procedure characteristics	Participant frequency	Percentage (%)
Type of most recent procedure (n = 98)		
Blood draw	15	15.3
Changing dressing over a wound	2	2.0
Heel prick	1	1.0
Imaging for potential fracture or dislocation	6	6.1
IV/catheter insertion	3	3.1
Nasal or throat swab (eg, coronavirus testing)	10	10.2
Needle injection (eg, vaccination, steroid, or insulin injection)	55	56.1
Stitches	1	1.0
Other procedure	5	5.1
When this procedure took place (n = 98)		
In the past week	3	3.1
In the past month	21	21.4
1–6 mo ago	41	41.8
6 mo to 1 y ago	17	17.4
More than 1 y ago	16	16.3
Setting of this procedure (n = 98)		
Home	3	3.1
Community setting (eg, at school)	2	2.0
Doctor's office or health clinic not at a hospital	37	37.8
Dentist office	3	3.1
Emergency department or urgent care	9	9.2
In hospital during a scheduled appointment (ie, outpatient)	20	20.4
In hospital during a stay (ie, inpatient)	4	4.1
Lab	2	2.0
Pharmacy	7	7.1
Public health unit	10	10.2
Present with the child during this procedure (n = 98)		
Yes	91	92.9
No	7	7.1
Who managed your child's pain during this procedure* (n = 91)		
Healthcare provider	38	41.8
Other family member	1	1.1
Parent/caregiver (yourself or a different caregiver)	56	61.5
They managed their own pain	12	13.2
No one	6	6.6
Strategies used during this procedure* (n = 91)		
Psychological		
Asking child to rate pain	4	4.4
Distraction	50	55.0
Encouraging child to be mindful and attend to the present	4	4.4
Encouraging child to pace their activities	5	5.5
Encouraging child to reach out to friends for support	1	1.1
Encouraging relaxation	54	59.3
Giving child something to look forward to	21	23.1
Helping child use imagination to relax	4	4.4
Letting child stay home from school	4	4.4
Talking about pain management strategies	16	17.6
Talking of unrelated things	39	42.9
Using technology to distract	18	19.8
Physical		
Massaging	7	7.7
Positioning in a way to minimize pain	19	20.9
Skin to skin contact	11	12.1
Using cold or heat	4	4.4
Pharmacological		
Breastfeeding	2	2.2
Numbing creams	3	3.3
Over the counter analgesics	13	14.3
Sugar water	1	1.1
Not listed	1	1.1
I did not use any strategies	1	1.1
Healthcare provider and pain management* (n = 98)		
Healthcare provider gave pain management information before the appointment	20	20.4
Healthcare provider gave pain management information right before or during the procedure	29	29.6
Healthcare provider gave pain management information after the procedure	24	24.5
Healthcare provider gave support to my child	19	19.4
Healthcare provider asked if I had any questions or concerns about my child's pain	16	16.3
Healthcare provider gave information without being asked	11	11.2
Healthcare provider gave information after being asked	4	4.1
Healthcare provider did not give any information about pain management	24	24.5
I don't remember	8	8.2

* Participants could select multiple responses.

**Figure 1.**
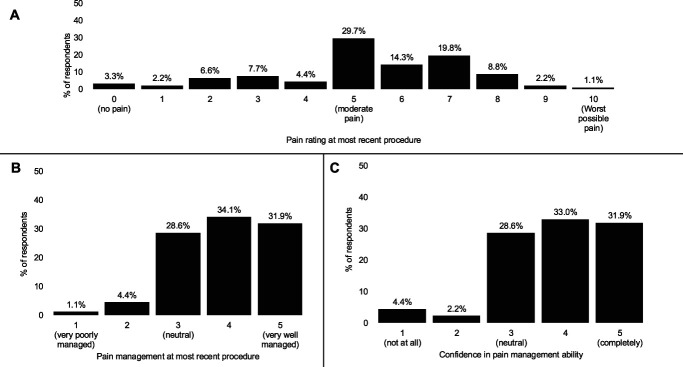
Bar charts illustrating the caregivers' perceptions of their child's most recent procedure (n = 91)* (A) 0–10 scale of their child's pain; (B) Likert-type scale of how well they felt their child's pain was managed; (C) Likert-type scale of their confidence in their ability to help manage their child's pain. *n = 91 is generated because respondents who selected that they did not remember their child's last procedure or were not present at the procedure were made to skip these questions.

**Figure 2.**
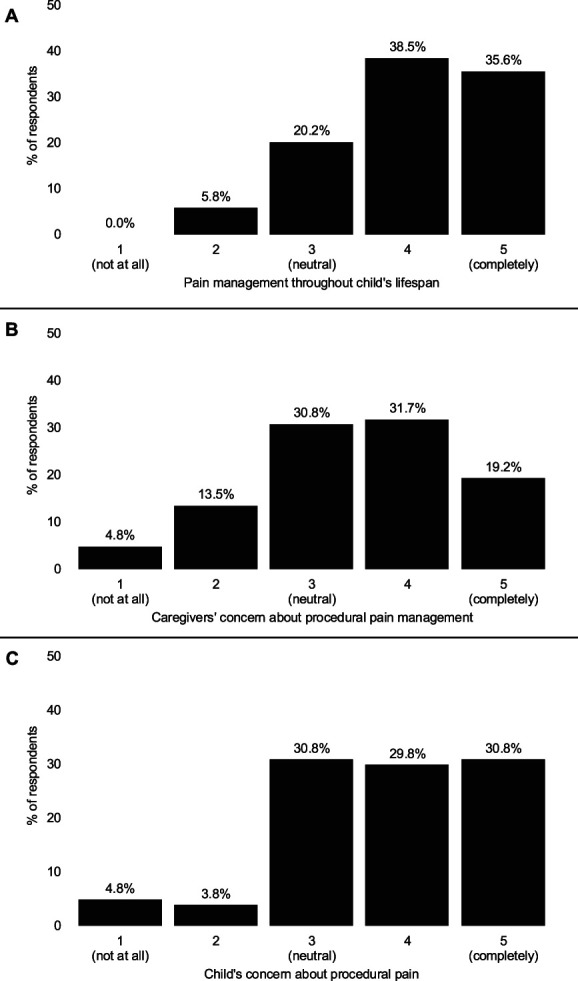
Bar charts illustrating the caregivers' perception of their child's procedural pain considering all past procedures using Likert-type scales (n = 104) (A) their broad rating of how well their child's procedural pain has been managed; (B) their rating of their overall concern about their child's procedural pain management; (C) their rating of their child's concern about procedural pain.

Caregivers' perceptions of their child's most recent procedure are presented in Figure 1. Caregivers' broad perceptions of their child's procedural pain experiences throughout their lifespan are presented in Figure 2.

Based on the Mann–Whitney *U* test, the caregivers' pain management ratings differed based on certain child demographics. Caregivers with male children rated their child's procedural pain management throughout their lifespan higher (mean rank = 59.18) than caregivers with female children (mean rank = 46.31; *P* < 0.05), but there was no significant difference between these groups in their rating of their child's most recent procedural pain management. Caregivers with children without a fear of needles rated their child's pain management at their most recent procedure (mean rank = 52.36) and all throughout their lifespan higher (mean rank = 59.10) than those whose children had a fear of needles at their most recent procedure (mean rank = 40.55; *P* < 0.05) and throughout their lifespan (mean rank = 45.90; *P* < 0.05). Caregivers with children who did not have a history of hospitalization rated their child's pain management throughout their lifespan higher (mean rank = 58.16) than those whose children had a history of hospitalization (mean rank = 43.06; *P* < 0.01), but there was no significant difference between these groups and their rating of their child's most recent procedural pain management. There were no significant differences in pain management experience regarding caregiver's sex or age, their child's age, the procedure setting, or the procedure type.

When caregivers were asked about their experience with information or tools for procedural pain management, 70.2% reported satisfaction with the tools and information available to them to help manage their child's pain as ≥4/5 (1 = *not at all* to 5 = *completely*). Overall, 42.9% of caregivers did not seek out information on how to manage their child's procedural pain. Of those who did, 36.7% sought it from electronic sources (eg, apps, search engines, websites, video sites, social media), 34.7% from their healthcare providers, and 29.6% from peers or themselves (eg, friends, family, prior experience). Caregivers reported that less than a quarter (24.5%) of healthcare providers did not provide any information on pain management and 4.1% gave information only after being asked.

For those who did seek out information or tools, 57.1% of caregivers rated their helpfulness and accessibility (ie, easy to find and/or access the information) at ≥4/5 and 62.2% rated their ease of understanding the information at ≥4/5 (1 = *not at all* to 5 = *completely*). Based on the Spearman Rho test, there was a significant positive correlation between the confidence caregivers had in their pain management ability and the helpfulness (r = 0.489, *P* < 0.001), accessibility (r = 0.384, *P* = 0.001), and ease of understanding (r = 0.330, *P* = 0.005) of the information they accessed. Based on the Mann–Whitney *U* test, there was a significantly higher rating of helpfulness of the information among male caregivers (mean rank = 46.27) then female caregivers (mean rank = 36.11; *P* < 0.05). There were no significant differences in responses on the helpfulness, accessibility, or understanding of the information caregivers sought out based on caregiver or child age, child sex, procedure setting, or procedure type. There were 18.3% of caregivers who reported previous awareness of tools designed to help them manage their child's procedural pain. Those who reported awareness of such tools were asked to describe them, and they identified videos, search engines, and brochures using “YouTube,” “Internet,” and “brochures” as descriptors in open-ended responses. No specific videos, websites, or brochures were identified.

### 3.3. Barriers to helping with procedural pain management

In open-ended responses, caregivers reported on the challenges they experience when helping their child manage procedural pain, identifying 3 main areas: caregiver-related, child–caregiver interaction-related, and healthcare professional-related barriers.

#### 3.3.1. Caregiver-related barriers

Caregiver-related barriers were identified as barriers experienced by the caregiver themselves. This was categorized into 4 subthemes: (1) pharmaceutical concerns, (2) lack of knowledge, (3) caregiver's own emotions, and (4) absence. Caregivers were worried about pharmaceutical use, raising concerns around fear of adverse effects and overuse. One caregiver indicated that they “feared that if too strong pain medication is used, the child will get addicted.” Caregivers also expressed a lack of knowledge and available information about pain management strategies and how to apply them for relevant procedures. Caregivers indicated that their own emotions, such as stress and fear, are a barrier they face when helping their children manage their pain. One caregiver said, “I am too emotional which makes her nervous, so her dad is usually the one to go in.” Finally, some caregivers indicated that they were unable to be present when their children underwent painful procedures, thus unable to help manage their child's pain in that moment.

#### 3.3.2. Child–caregiver interaction-related barriers

Child–caregiver interaction-related barriers were identified as challenges related to the child and caregiver relationship. This was divided into 2 subthemes: (1) communication challenges, and (2) management of children's emotions. Caregivers indicated challenges communicating with the child about the procedure, with one caregiver saying their child is “too young to understand and not able to communicate.” Other caregivers indicated that their child's emotions during the procedure were hard to manage. One participant reported that their child “just gets uncontrollable[,] screaming and not cooperating.”

#### 3.3.3. Healthcare professional-related barriers

Healthcare professional-related barriers were identified as challenges related to healthcare professionals. Caregivers suggested that the attitudes of healthcare professionals presented a barrier to pain management with one indicating that the “[doctor] or nurses don't care about pain management for kids because they just want the procedure done.”

## 4. Discussion

Children commonly experience pain from medical procedures from infancy to adolescence. Despite established best practices, this type of pain often remains undertreated. Experts emphasize the importance of families taking an active role in procedural pain management.^28,33,38^ Overall, this study found that most caregivers are present during their children's short painful medical procedures and while most report satisfaction with the current management of their child's pain, there is still a proportion of caregivers who say that their child's pain was not well-managed. The results also reveal that although many caregivers report playing an active role in their child's procedural pain management, they face challenges when it comes to doing so.

Despite many caregivers' reporting satisfaction with the current state of pediatric procedural pain management, there are some that continue to be dissatisfied and many that remain concerned about their child's pain. Determining the clinical significance of pain is a difficult concept; however, it can be suggested that most children in this study experienced clinically significant pain during their most recent medical procedure, as previous pediatric studies have reported that pain rated >3/10 typically influences a child's function.^5^ This discrepancy between children's clinically significant pain experience, caregivers' concern about this pain, and their general satisfaction with pain management, may suggest that some caregivers are not aware of the possibilities for better procedural pain control available. Alternatively, where some caregivers continue to rate their child's pain management low, this may reflect unreasonable expectations for what constitutes well-managed pain. Even best practice strategies cannot eliminate the pain associated with these procedures, and it is unclear whether this is understood by all caregivers. Thus, these results suggest that there remains work to be performed to improve pediatric pain management overall, in particular improving caregivers' awareness of best practices.

Efforts are being made by caregivers to help children manage their pain during procedures, with most reporting they played a role in procedural pain management and few reporting that no pain management strategies were used. In addition, most caregivers were confident in their ability to help their child with procedural pain management; however, the quality and effectiveness of these efforts are unclear. Many caregivers reported using evidence-based techniques, such as distraction. However, despite the best practice that procedural pain management incorporate psychological, physical, and pharmacological components,^38^ only a subset of caregivers reported using pharmacological strategies. Based on the qualitative responses, this may stem from a lack of knowledge on the caregiver's part and a concern around the side effects of medications. In addition, over half of the caregivers reported using reassurance (eg, saying “this won't hurt”) as a strategy, which has been linked to the worsening of a child's perception of pain.^22^ Therefore, there needs to be continued effort to educate caregivers on the strategies available, their safety profiles and their efficacy, with the aim of supporting caregivers use of or advocacy for these strategies to improve their child's procedural pain experience.

Potential reasons for the pain management dissatisfaction and the lack of active participation in pain management among some caregivers may be linked to the perceived barriers caregivers identified related to helping with pain management. The major barriers discussed included their own lack of knowledge, their child's behaviour and emotions, and healthcare professional's attitudes. These barriers overlap with those previously described by healthcare providers and parents.^1,14,17^ Caregivers reported that less than half of the healthcare providers played a role in procedural pain management correlating to the qualitative data in which caregivers perceived a lack of value placed on procedural pain management by healthcare providers. This aligns with prior reports from healthcare providers highlighting the “culture” of emergency departments as a barrier to implementing pain management best practices.^1^ Some of the perceived barriers, such as lack of knowledge and concerns about pharmaceuticals, could potentially be addressed by knowledge mobilization interventions (KMIs). The perceived barrier of healthcare professional attitudes may require an approach that integrates both KMIs that target individuals and systems-level changes that make it easier for providers to prioritize pain management in busy clinical settings. These findings suggest that addressing pediatric procedural pain requires a multifaceted approach that includes both caregivers and healthcare providers.

While some caregivers sought information on procedural pain management and were aware of KMIs for pediatric pain management, none provided specific examples, suggesting the need for improved knowledge mobilization efforts around procedural pain management. Caregivers are currently seeking procedural pain information from a variety of sources with a predominance toward seeking information from electronic sources, healthcare providers, and peers. Caregivers' confidence in their ability to help manage pain was related to their use of tools they found helpful, accessible, and easy to understand. This suggests the potential for caregiver-targeted KMIs to empower them to take an active role in procedural pain management and improve the implementation of procedural pain management best practices. This is supported by existing caregiver-targeted KMIs that have improved the implementation of vaccination pain management.^7,37^ Interestingly, male caregivers rated the information currently available on pain management more helpful than female caregivers, a difference which to our knowledge has not previously been described.

### 4.1. Strengths and limitations

This study provides insights into caregiver's perceptions, knowledge, and behaviours related to pediatric procedural pain management and the barriers they face when attempting to help with this. Where much previous research studying pediatric procedural pain had a predominance of female participants,^11–14,19,21,26^ a strength of this study is that nearly half of respondents were male, providing a more sex-balanced perspective than previously seen. Although an attempt was made to solicit diverse respondents in other areas, such as race and age, future research with a larger and more diverse sample may support the generalizability of the present findings. Another limitation was the predominance of outpatient settings as the location of the most recent procedure caregivers were asked to consider. As a result, other settings where painful procedures commonly occur (eg, emergency department and inpatient units) were underrepresented. This may limit the present findings as the resources available and protocols vary widely across clinical settings, potentially affecting caregivers' perspectives and behaviours. In addition, the data were based on self-report survey responses of caregivers, which is subject to biases and selective reporting, particularly with their recall of past events and their ability to accurately characterize their child's pain experience. For example, it is possible that more healthcare providers helped with pain management than was reported but that this was not observed or recollected by respondents. However, because the primary aim of this study was to determine caregivers' perceptions, a subjective judgment by nature, the influence of bias is unlikely to change the present findings that participants perceive ongoing barriers to them helping with procedural pain management.

## 5. Conclusion

Overall, this study highlighted that while most caregivers are present during their children's procedural pain, there is room to further support them in playing an active role in procedural pain management, which could improve the implementation of best practices and more positive immediate- and long-term health outcomes for children. While there is general satisfaction with current pain management practices among caregivers, there remains room for improvement. Initiatives to mitigate some of the barriers to helping with pain management perceived by caregivers, such as their own lack of knowledge, their distress, and their child's distress, may lead to improved procedural pain management for children.

## Disclosures

The authors have no conflict of interest to declare.
